# Ventilator-induced lung injury is aggravated by antibiotic mediated microbiota depletion in mice

**DOI:** 10.1186/s13054-018-2213-8

**Published:** 2018-10-29

**Authors:** Sandra-Maria Wienhold, Mario Macrì, Geraldine Nouailles, Kristina Dietert, Corinne Gurtner, Achim D Gruber, Markus M Heimesaat, Jasmin Lienau, Fabian Schumacher, Burkhard Kleuser, Bastian Opitz, Norbert Suttorp, Martin Witzenrath, Holger C Müller-Redetzky

**Affiliations:** 1 Division of Pulmonary Inflammation, Charité – Universitätsmedizin Berlin, corporate member of Freie Universität Berlin, Humboldt-Universität zu Berlin, and Berlin Institute of Health, Berlin, Germany; 2 Department of Veterinary Pathology, Freie Universität Berlin, Berlin, Germany; 3 Institute for Microbiology and Infection Immunology, Charité – Universitätsmedizin Berlin, corporate member of Freie Universität Berlin, Humboldt-Universität zu Berlin, and Berlin Institute of Health, Berlin, Germany; 4 Department of Nutritional Toxicology, Institute of Nutritional Science, University of Potsdam, Nuthetal, Germany; 5 Department of Molecular Biology, University of Duisburg-Essen, Essen, Germany; 6 Department of Infectious Diseases and Pulmonary Medicine, Charité – Universitätsmedizin Berlin, corporate member of Freie Universität Berlin, Humboldt-Universität zu Berlin, and Berlin Institute of Health, Charitéplatz 1, 10117 Berlin, Germany; 7 Present address: Scuola di specializzazione in Anestesia, Rianimazione e Terapia Intensiva, Università degli Studi di Milano, Milan, Italy; 8 Present address: Institute of Animal Pathology, Department of Infectious Diseases and Pathobiology, Vetsuisse Faculty, University of Bern, Bern, Switzerland

**Keywords:** Broad-spectrum antibiotic therapy, Ventilator-induced lung injury, Microbiota

## Abstract

**Background:**

Antibiotic exposure alters the microbiota, which can impact the inflammatory immune responses. Critically ill patients frequently receive antibiotic treatment and are often subjected to mechanical ventilation, which may induce local and systemic inflammatory responses and development of ventilator-induced lung injury (VILI). The aim of this study was to investigate whether disruption of the microbiota by antibiotic therapy prior to mechanical ventilation affects pulmonary inflammatory responses and thereby the development of VILI.

**Methods:**

Mice underwent 6–8 weeks of enteral antibiotic combination treatment until absence of cultivable bacteria in fecal samples was confirmed. Control mice were housed equally throughout this period. VILI was induced 3 days after completing the antibiotic treatment protocol, by high tidal volume (HTV) ventilation (34 ml/kg; positive end-expiratory pressure = 2 cmH_2_O) for 4 h. Differences in lung function, oxygenation index, pulmonary vascular leakage, macroscopic assessment of lung injury, and leukocyte and lymphocyte differentiation were assessed. Control groups of mice ventilated with low tidal volume and non-ventilated mice were analyzed accordingly.

**Results:**

Antibiotic-induced microbiota depletion prior to HTV ventilation led to aggravation of VILI, as shown by increased pulmonary permeability, increased oxygenation index, decreased pulmonary compliance, enhanced macroscopic lung injury, and increased cytokine/chemokine levels in lung homogenates.

**Conclusions:**

Depletion of the microbiota by broad-spectrum antibiotics prior to HTV ventilation renders mice more susceptible to developing VILI, which could be clinically relevant for critically ill patients frequently receiving broad-spectrum antibiotics.

**Electronic supplementary material:**

The online version of this article (10.1186/s13054-018-2213-8) contains supplementary material, which is available to authorized users.

## Background

Mechanical ventilation (MV) is a live-saving intervention in acute respiratory failure. Patients in intensive care units (ICU) are often subjected to MV, which can induce local and systemic inflammatory responses, thereby leading to the development of ventilator-induced lung injury (VILI) [1, 2]. VILI contributes to mortality and morbidity in patients with respiratory failure [1, 3, 4].

Critically ill patients in the ICU frequently receive antibiotics [5], and exposure to broad-spectrum antibiotics alters the microbiota. The microbiota is an “ecological community of commensal, symbiotic and pathogenic microorganisms” [6], which can be found e.g. in the gut, on the skin, and in the respiratory tract [7]. Recent studies revealed a significant impact of the physiological gut microbiota on the homeostasis of the immune system. The intestinal bacterial flora was shown to be essential for the calibration of local inflammatory responses of the gut [8]. Further, intact gut microbiota calibrates the systemic homeostasis between inflammatory and anti-inflammatory components and is essential for an effective immune response e.g. by altering IL-10 activity [9]. Recent studies suggest that the gut microbiota can also affect the pulmonary inflammatory response and associated lung injury [10–12]. For instance, lung inflammation, organ damage and mortality have been shown to be increased in antibiotic-treated mice infected with *Streptococcus pneumoniae* compared to controls, suggesting that the gut microbiota modulates local inflammatory responses in the lungs [13]. Translocation of commensal bacteria and their metabolites, including short-chain fatty acids, from the gut into the bloodstream was suggested as a potential underlying mechanism of the gut-lung interaction [14–16]. Moreover, Clarke et al. revealed that components of the microbiota, after translocation from the gut into the bloodstream, also regulate the inflammatory activity of neutrophilic granulocytes [16]. This might be helpful for the host in the case of infection, but might be harmful within the context of autoimmunity or tissue trauma-induced inflammation caused by e.g. MV.

Currently, little is known about the effect of the microbiota on local stimulation of the immune system and pulmonary inflammatory phenotype in sterile lung inflammation. Although great effort is made to minimize antibiotic exposure in general, certain groups of patients are exposed to long and frequent antibiotic treatment. This includes patients that are rendered immunosuppressed by e.g. chemotherapy and that are often treated with antibiotics more frequently, for longer periods and under certain circumstances even for prophylaxis. Hence, by depleting the microbiota by antibiotic treatment prior to MV we examined the impact of the microbiota on the pulmonary inflammatory response to MV and thereby its influence on the development of VILI. Some of the results presented here were previously reported in the form of abstracts [17–19].

## Methods

### Animals

Female C57BL/6N mice (Charles River, Sulzfeld, Germany) were used. Mice were housed under specific pathogen-free conditions with free access to food and water and 12 h light/dark cycle. Animal housing and experimental procedures complied with the Federation of European Laboratory Animal Science Associations (FELASA) guidelines and recommendations for the care and use of laboratory animals.

### Generation of microbiota-depleted mice

Long-term antimicrobial therapy was performed as previously described [20]. Briefly, 8-week-old mice were transferred to sterile cages and received a fivefold broad-spectrum antibiotic cocktail (ampicillin (1 g/l; Ratiopharm, Ulm, Germany), vancomycin (500 mg/l; Cell Pharm, Hannover, Germany), ciprofloxacin (200 mg/l; Bayer Vital, Leverkusen, Germany), imipenem (250 mg/l; MSD, Haar, Germany), and metronidazole (1 g/l; Fresenius, Bad Homburg, Germany)) via drinking water ad libitum for 6–8 weeks until the absence of cultivable bacteria in fecal samples was confirmed. Absence of cultivable bacteria in feces samples (applying thioglycolate enrichment broths; Oxoid, Wesel, Germany) for at least three consecutive weeks served as quality control for successful depletion of gut microbiota [20]. Applying the qPCR technique we observed that antibiotic therapy significantly depleted the commensal intestinal bacteria by 2–3 log levels compared to non-treated mice (Additional file 1: Figure S1). MV of depleted and control mice (ctrl) started 72 h after completing the antibiotic treatment protocol at the age of 14–15 weeks.

### Mechanical ventilation

MV was performed as previously described [21]. Mice were anesthetized with intraperitoneal injections of fentanyl (0.05 mg/kg), midazolam (5 mg/kg), and medetomidine (0.5 mg/kg). Repetitively, fentanyl (0.016 mg/kg), midazolam (1.6 mg/kg), and medetomidine (0.16 mg/kg) were supplied via an intraperitoneal catheter, when required, to guarantee adequate anesthesia during the experiment. Body temperature was maintained at 37 °C by a body temperature-controlled heating pad. Mice were tracheotomized, intubated, and ventilated with tidal volume of 8 ml/kg, respiratory rate of 160 per minute, fraction of inspiratory oxygen (FiO_2_) of 0.5 and 2 cmH_2_O positive end-expiratory pressure (PEEP). A carotid artery catheter was applied for blood pressure monitoring and infusion of a balanced electrolyte solution (Jonosteril; Fresenius, Bad Homburg, Germany) containing 150 M trometamol hydrochloride (B. Braun Melsungen AG, Melsungen, Germany) (350 μl/h).

Mice were ventilated using a special rodent ventilation system, which continuously recorded airway pressure, respiratory rate, and tidal volume. Dynamic elastance, resistance, and compliance were measured every 5 min using a forced oscillation technique (flexiVent; Scireq, Montreal, QC, Canada). Static compliance was recorded during the initial recruitment maneuver and at the end of the experiment after exsanguination.

After preparation, a recruitment maneuver was performed (increasing airway pressure to 27 cmH_2_O), and mice were ventilated for 4 h with the ventilator settings according to the study groups:

1. Low tidal volume (LTV): tidal volume of 8 ml/kg, respiratory rate of 160/min; deep inflation (27 cmH_2_O for 1 s) was performed every 5 min to avoid atelectasis.
2. High tidal volume (HTV): tidal volume of 34 ml/kg, respiratory rate of 70/min.
3. Non-ventilated (NV): ctrl mice, LTV ventilation was stopped after 10 min to generate baseline data on MV.

To avoid differences in pH value and partial arterial pressure of carbon dioxide (paCO_2_) the dead space of the respirator tubing was adjusted in the HTV groups resulting in equal values in both groups.

Mean blood pressure, heart rate, and peripheral oxygen saturation (MouseOx; Starr Life Science Corp., Oakmont, PA, USA) were recorded every 10 min during the experiment. In all groups, mean arterial pressure remained stable during the experiment indicating that a potential influence of haemodynamic deterioration on the results can be excluded (Additional file 1: Figure S2).

Ninety minutes before the end of experiment, 1 mg human serum albumin (HSA) was administered through the arterial catheter. After 240 min of MV, FiO_2_ was increased to 1.0 for 10 min. Five minutes before final exsanguination, heparin was administered through the arterial catheter. At termination of the experiment, mice were killed by exsanguination via the carotid artery catheter. Two LTV ctrl and one LTV antibiotic-treated (ATB) mice had to be excluded due to development of pneumothorax during ventilation. One LTV and two HTV antibiotic-treated mice, two HTV ctrl mice, one NV ctrl, and one NV antibiotic mouse had to be excluded due to technical issues during the experiment. One LTV antibiotic-treated mouse had to be euthanized during antibiotic treatment. The resulting number in each group were: NV ctrl and NV ATB *n* = 9, LTV ctrl *n* = 8, LTV ATB *n* = 7, HTV ctrl and HTV ATB *n* = 8. For histological examination three mice were examined in each group. For the detailed numbers of animals per figure please refer to the figure legends.

### Blood gas analysis

Blood samples were immediately analyzed using a blood gas analyzer (ABL 800; Radiometer, Copenhagen, Denmark) to determine partial pressure of arterial oxygen (PaO_2_). The oxygenation index (OI) was calculated as the product of mean airway pressure and the FiO_2_ divided by PaO_2_ as described previously [21].

### Differential blood count and plasma preparation

Blood leukocytes and platelets were differentiated using a Scil Vet abc hematology analyzer (Scil animal care company GmbH, Viernheim, Germany). Remaining blood was centrifuged (12000 × g, 10 min) and plasma stored at − 80 °C.

### Histopathologic analysis

After exsanguination, the lungs were photographed. Subsequently, the lungs were carefully removed and fixed in formalin pH 7.0 for 24–48 h, embedded in paraffin and cut into 2-μm sections. After routine dewaxing and dehydration, sections were stained with hematoxylin and eosin (H&E) or Periodic acid-Schiff reaction (PAS). Organs were microscopically evaluated by two independent board-certified veterinary pathologists, blinded to the study groups. Due to red cast reduction of macro images, color auto-correction using ADOBE® PHOTOSHOP® CS5 Extended, Version 12.0.4 was performed.

### Bronchoalveolar lavage and lung processing

The lungs were perfused with sterile cooled 1 × PBS (Gibco; Thermo Fisher Scientific, Altham, MA, USA) via a catheter ligated in the pulmonary artery. Prior to bronchoalveolar lavage (BAL) of the left lungs, the right stem of the bronchus was ligated. Lavage was performed four times with 400 μl PBS containing protease inhibitor (complete mini; MERCK KGaA, Darmstadt, Germany). BAL fluid (supernatant) and BAL cells (pellet) were separated by centrifugation at 300 × g. BAL fluid was shock-frosted in liquid nitrogen and stored at − 80 °C until further analysis. BAL cells were resuspended in staining buffer and stored at 4 °C. The right lung lobes were minced, divided into three, two parts were shock-frosted in liquid nitrogen and stored at − 80 °C until further analysis. One part was used for lung leukocyte isolation.

### Isolation of lung leukocytes and flow cytometry

For isolation of lung leukocytes, minced lung tissue was digested in RPMI medium supplemented with collagenase (MERCK KGaA) and DNAse (PanReack AppliChem GmbH, Darmstadt, Germany) for 30 min at 37 °C. Single cell suspension was prepared by using 70-μm cell strainers (BD, Heidelberg, Germany) and red blood cell lysis was performed. BAL and lung immune cells were differentiated by flow cytometry. Isolated BAL cells were blocked with anti-CD16/CD32 (BD) and stained with anti-CD11c (N418; ATCC), anti-CD11b (M1/70; eBioscience, Vienna, Austria), anti-F4/80 (BM8; eBioscience), anti-Ly6G (1A8; BD), and anti-Ly6C (AL-21; BD) monoclonal antibodies (mAbs). The FL-1 channel was used to determine autofluorescence of cells. The gating strategy is shown in Additional file 1: Figure S3. Isolated lung cells were blocked with anti-CD16/CD32 (BD), surface stained with anti-CD4 (RM4–5; BD), anti-CD8 (53–6.7; eBioscience), anti CD3 (17A2; eBioscience), and gdTCR (GL-3; BD), fixed and permeabilized with FoxP3 / Transcription Factor Staining Buffer Set (eBioscience) and stained intranuclearly with anti-FoxP3 (FJK-16s; eBioscience) mAbs. All stained cells were acquired using a BD FACS Canto II and analyzed with BD FACSDiva and FlowJo software. For calculation of total cell numbers, CountBright Absolute Counting Beads (Thermo Fisher Scientific) were used.

### Lung permeability

The HSA concentration was determined in BAL fluid (BALF) and plasma by ELISA (Bethyl, Biomol, Hamburg, Germany), and the HSA BALF/plasma ratio was calculated to assess the alveolar-capillary barrier permeability index as described previously [22–24].

### Cytokine protein measurements

Prior to analysis, minced lung tissues were pre-treated with protein lysis buffer (BD). Cytokines were quantified from plasma and lung tissue samples by ProcartaPlex Custom Mix & Match according to the manufacturer’s instructions (eBioscience).

### mRNA isolation and expression analysis

Minced lung tissue was homogenized with Gentle MACS M tubes (Miltenyi Biotec, Bergisch Gladbach, Germany) in TRIzol and RNA was extracted according to the manufacturer’s instructions (Thermo Fisher Scientific). cDNA was transcribed by high capacity reverse transcription kit (Applied Biosystems, Life Technologies, Darmstadt, Germany). Quantitative PCR (qPCR) was performed on ABI 7300 using TaqMan gene expression assays (Applied Biosystems, Life Technologies). The qPCR conditions were as follows: denaturation in one cycle of 2 min at 50 °C and 10 min at 95 °C, followed by 40 cycles of 15 s at 95 °C, 20 s at 60 °C, and 1 min at 72 °C. Results were normalized to *Gapdh* levels and compared to the average expression of the target signals in the NV control mice as described previously [21, 25]. Primer and probe sequences are provided in Additional file 1: Table S1.

### Molecular analysis of fecal microbiota

Fecal pellets were collected directly before ventilation and stored at − 80 °C until measurement. DNA was extracted as described previously [20, 26, 27]. Bacteria, which are mainly found in the gut of mice were examined using qPCR with species-specific, genera-specific or group-specific 16S rRNA gene primers (Tib MolBiol, Berlin, Germany) as described previously [28]. Briefly, numbers of 16S rRNA gene copies per nanogram DNA were assessed including total eubacterial load, *Enterococcus spp*., lactic acid bacteria, *Bifidobacterium spp.*, *Bacteroides/Prevortella spp*., *Clostridium coccoides* group, *Clostridium leptum* group and mouse intestinal *Bacteroides*.

### Data analyses

Data are expressed as mean with SD or boxplots depicting median, quartiles, and range excluding outliers (as shown as open circles) or as single values with median. For comparison between two groups the Mann-Whitney U test was used. For grouped analyses, two-way analysis of variance (ANOVA) or two-way-repeated measures ANOVA/Tukey’s multiple comparison test was performed. *P* values < 0.05 were considered significant. Analyses were performed using GraphPad Prism 6 (San Diego, CA, USA).

## Results

### Microbiota depletion prior to mechanical ventilation aggravated HTV ventilation-induced worsening of lung function

**Fig. 1:**
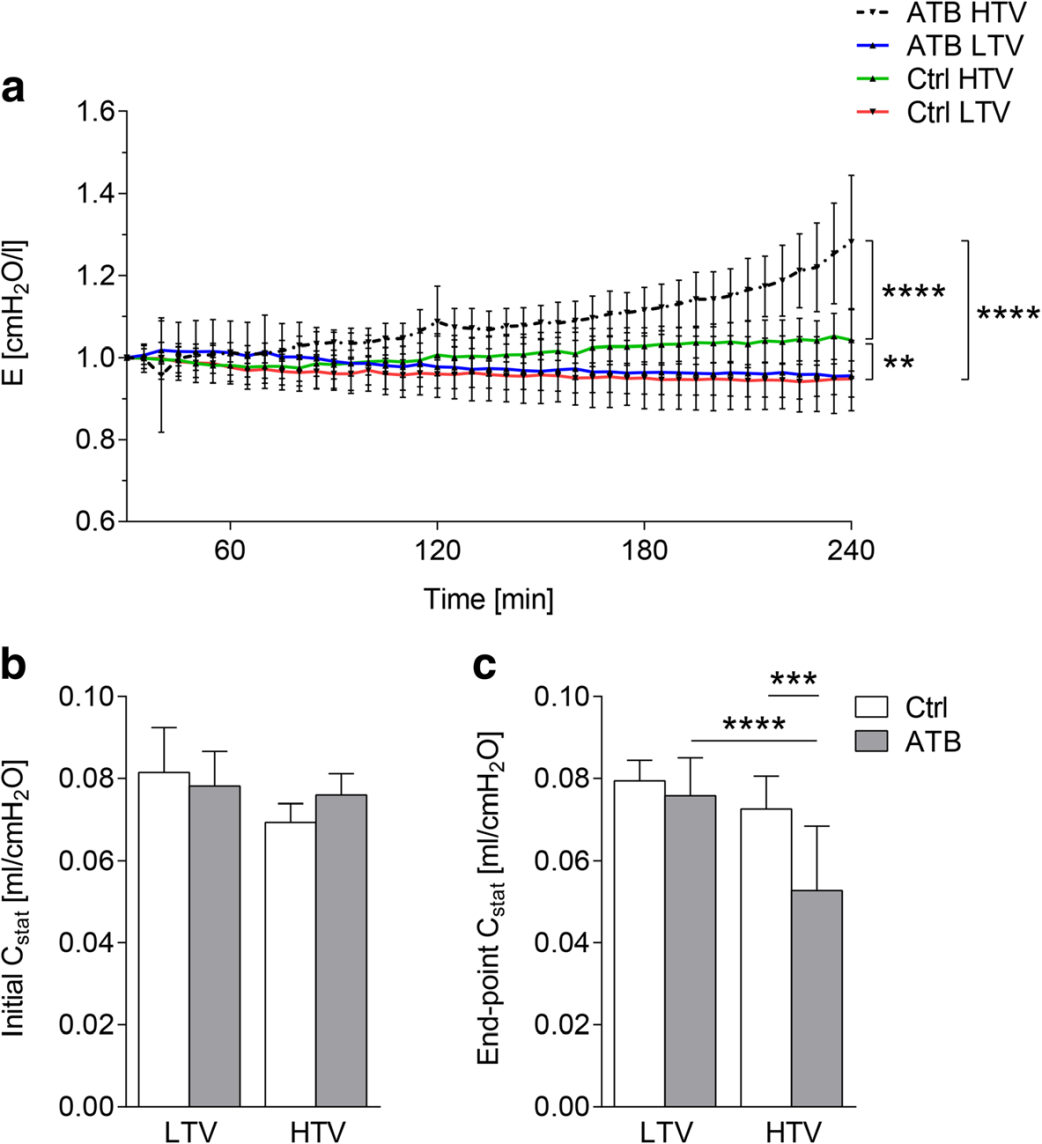
Microbiota depletion prior to mechanical ventilation aggravated high tidal volume (HTV) ventilation-induced worsening of lung function. Mice received antibiotic broad-spectrum therapy (ATB). Control (Ctrl) mice did not receive antibiotic treatment. Ventilator-induced lung injury (VILI) was induced 3 days after completing antibiotic treatment protocol by (HTV) ventilation (34 ml/kg; positive end-expiratory pressure = 2 cmH_2_O, 4 h). Additional groups of mice were ventilated with low tidal volume (LTV). **a** Dynamic elastance (E) was measured in 5-min intervals during ventilation. Baseline was set to 30 min and values were normalized to baseline. **b**-**c** Static compliance (C_stat_) was measured at the start (**b**) and after 4 h of ventilation (**c**). Values are given as mean ± SD (**a**) or mean and SD (**b**-**c**). In **a** *n* = 11 (LTV ctrl) or *n* = 7 (HTV ctrl, HTV ATB) or *n* = 6 (LTV ATB); in **b** *n* = 11 (LTV ctrl, HTV ctrl, HTV ATB) or *n* = 10 (LTV ATB); **c** *n* = 11 (HTV ctrl) or *n* = *10* (LTV ctrl, LTV ATB, HTV ATB). Analysis was by two-way repeated measures analysis of variance (ANOVA)/Tukey’s multiple comparison test (**a**) or by two-way ANOVA/Tukey’s multiple comparison test (**b**-**c**). **P < 0.01, ***P < 0.001, ****P < 0.0001

To investigate the impact of antibiotic-treatment-related disruption of the microbiome on ventilator-induced lung injury we measured parameters of lung function. Ventilation of mice with HTV led to an increase in pulmonary dynamic elastance compared to LTV ventilation, which was significantly higher following microbiota depletion (Fig. 1a). In contrast, in both groups ventilated with LTV, pulmonary dynamic elastance remained unaffected throughout the experiment.

The static compliance at the beginning of the ventilation showed no difference within the groups (Fig. 1b), whereas the static compliance was reduced in microbiota-depleted HTV-ventilated mice, compared to control mice after 4 h of ventilation (Fig. 1c). These data indicate that microbiota depletion by antibiotic treatment prior to MV worsens VILI.

### Microbiota depletion prior to MV increased pulmonary permeability and OI in HTV-ventilated mice

**Fig. 2:**
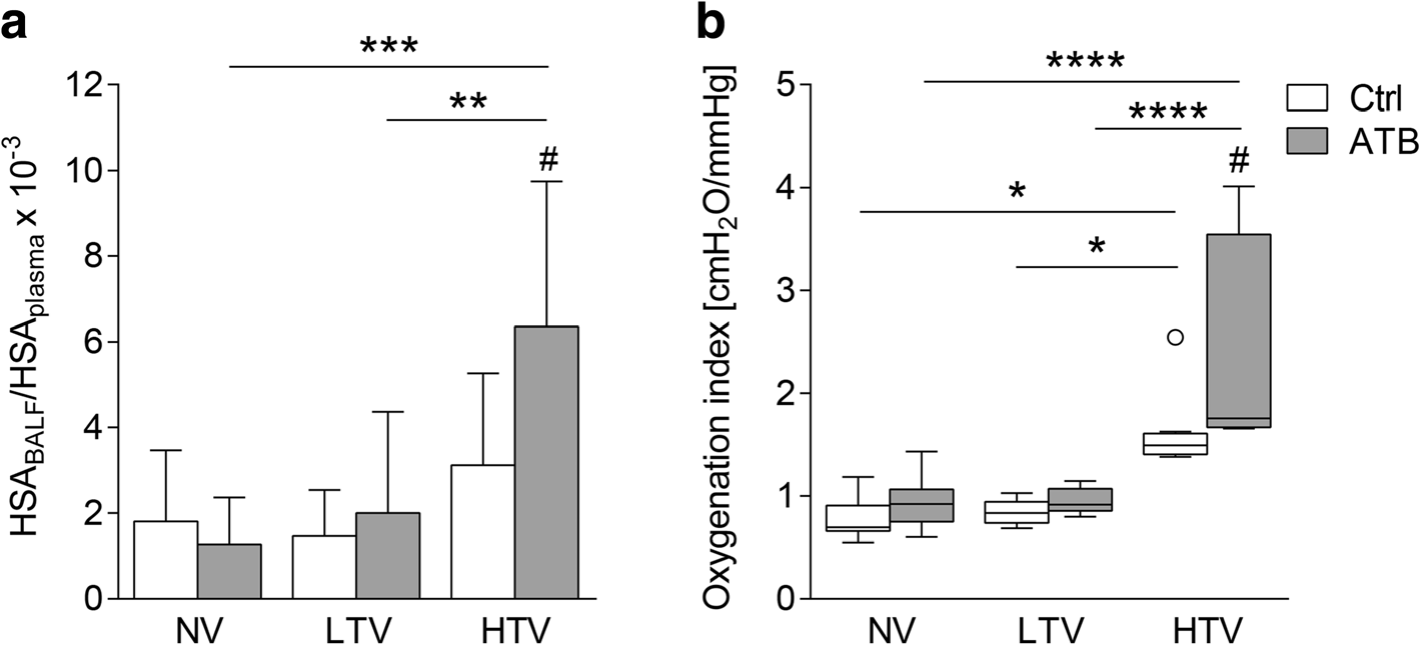
Microbiota depletion prior to mechanical ventilation increased pulmonary permeability and oxygenation index in high tidal volume (HTV)-ventilated mice. Mice received antibiotic broad-spectrum therapy (ATB). Control (Ctrl) mice did not receive antibiotic treatment. Ventilator-induced lung injury (VILI) was induced 3 days after completing antibiotic treatment protocol by (HTV) ventilation (34 ml/kg; positive end-expiratory pressure = 2 cm H_2_O, 4 h). Additional groups of mice were ventilated with low tidal volume (LTV) or did not receive ventilation (NV). **a** Pulmonary barrier integrity index was calculated as ratio of human serum albumin (HSA) protein levels determined by ELISA in bronchoalveolar lavage fluid (BALF) and plasma. **b** Oxygenation index (OI) was calculated as the product of mean airway pressure and the fraction of inspired oxygen divided by the partial pressure of arterial oxygen. Values are given as mean and SD (**a**) or box plots depicting median, quartiles and range excluding outliers (open circles) (**b**). In **a** *n* = 8 (NV ctrl, NV ATB, LTV ctrl, HTV ctrl, HTV ATB) or *n* = 6 (LTV ATB); in **b** *n* = 9 (NV ctrl) or *n* = 8 (LTV ctrl, HTV ctrl, NV ATB, HTV ATB), or *n* = 6 (LTV ATB), two-way analysis of variance/Tukey’s multiple comparison test, ^#^indicates difference in HTV ATB vs. HTV Ctrl, *as indicated, *^/#^*P* <0.05, **P < 0.01, ***P < 0.001, **** *P* < 0.0001

Since pulmonary hyperpermeability is the main underlying mechanism of edema formation and related worsening of lung function in acute lung injury, we measured pulmonary permeability. Microbiota depletion by antibacterial therapy prior to ventilation did not affect lung permeability in NV and LTV-ventilated mice. Non-treated HTV mice had a trend towards increased permeability compared to non-treated NV and LTV mice. Under HTV ventilation, pulmonary vascular permeability was significantly increased in mice after receiving antibacterial therapy (Fig. 2a). In the NV and LTV groups, the OI was unchanged throughout the experiment, while HTV-ventilated mice had a higher oxygenation index compared to NV and LTV-ventilated mice (Fig. 2b). In microbiota-depleted mice, HVT ventilation increased the OI significantly.

### Microbiota depletion prior to MV worsened lung injury in HTV-ventilated mice

**Fig. 3:**
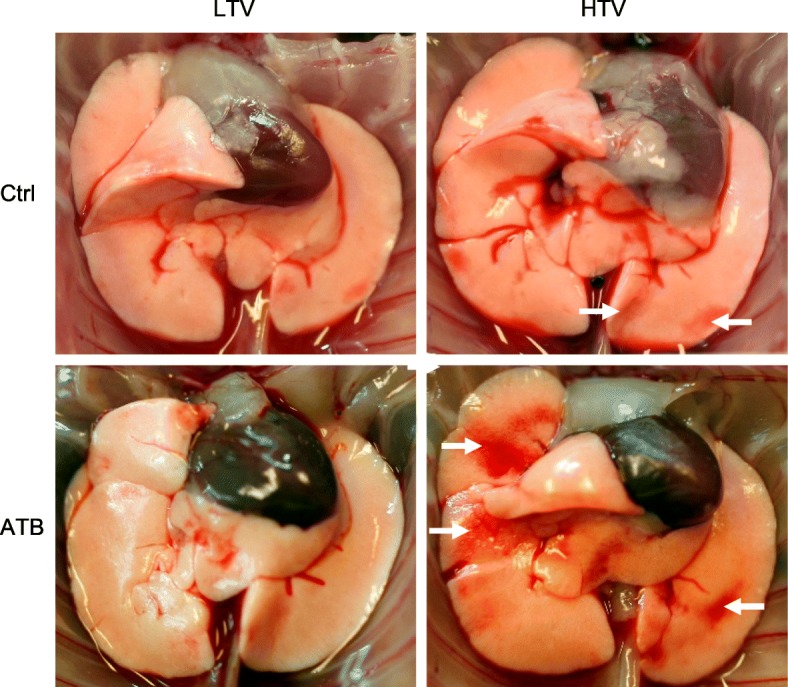
Microbiota depletion prior to mechanical ventilation worsened lung injury in high tidal volume (HTV)-ventilated mice. Mice received antibiotic broad-spectrum therapy (ATB). Control (Ctrl) mice did not receive antibiotic treatment. Ventilator-induced lung injury was induced 3 days after completing antibiotic treatment protocol by (HTV) ventilation (34 ml/kg; positive end-expiratory pressure = 2 cmH_2_O, 4 h). Additional groups of mice were ventilated with low tidal volume (LTV). Representative photographs of lungs after 4 h of ventilation with macroscopic lung lesions (reddish discolored areas (arrows)) are shown

The extent of ventilation correlates with lung tissue damage [21]. While ventilation with LTV did not lead to any macroscopic alteration of the lungs, HTV-ventilated mice had reddish discolored areas, which were more pronounced in mice receiving antibiotic therapy prior to mechanical ventilation (Fig. 3). No signs of acute lung injury were observed in naive mice (Additional file 1: Figure S4).

### Microbiota depletion prior to mechanical ventilation aggravated the inflammatory response in VILI

**Fig. 4:**
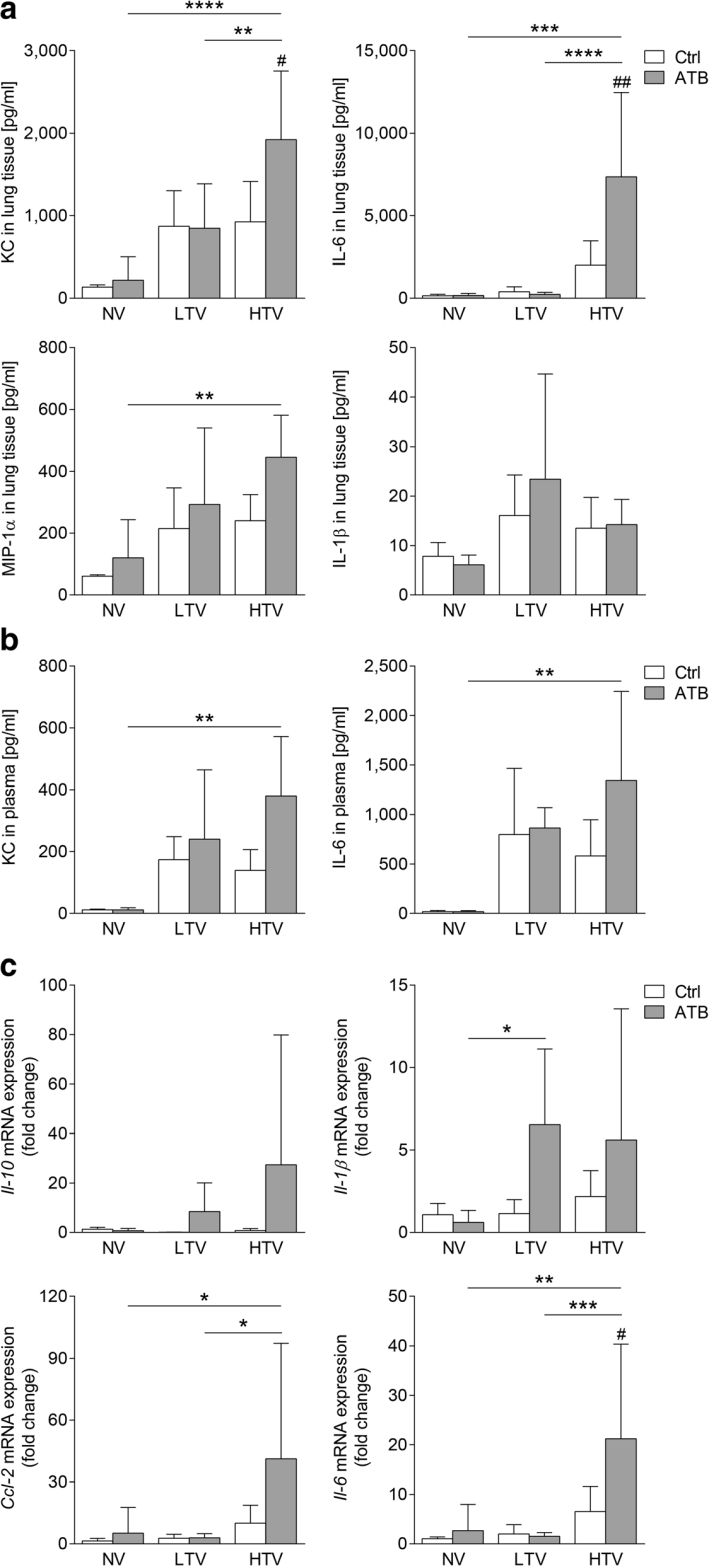
Microbiota depletion prior to mechanical ventilation aggravated the inflammatory response in ventilator-induced lung injury (VILI). Mice received antibiotic broad-spectrum therapy (ATB). Control (Ctrl) mice did not receive antibiotic treatment. VILI was induced 3 days after completing antibiotic treatment protocol by high tidal volume (HTV) ventilation (34 ml/kg; positive end-expiratory pressure = 2 cmH_2_O, 4 h). Additional groups of mice were ventilated with low tidal volume (LTV) or did not receive ventilation (NV). Keratinocyte-derived chemokine (KC), macrophage inflammatory protein-1α (MIP-1α), IL1β and IL-6 protein levels were measured in lung homogenates (**a**) and in plasma (**b**) (KC and IL-6) using multiplex assays. **c** mRNA expression levels of *Il-6*, *Il-10*, C*cl-2* and *Il-1β* were measured by qPCR, normalized to *Gapdh* levels and related to the average expression of the target gene in NV control mice as described previously [25]. Values are given as mean and SD. In **a** *n* = 8 (HTV ctrl), *n* = 7–8 (LTV ctrl), *n* = 7 (HTV ATB, LTV ATB), *n* = 5 (NV ATB), or *n* = 4 (NV ctrl); in **b** *n* = 6–8 (HTV ATB), *n* = 6–7 (LTV ctrl, HTV ctrl), *n* = 5 (NV ATB), *n* = 4–7 (LTV ATB), or *n* = 3 (NV ctrl); in **c** *n* = 8 (NV ATB), *n* = 6–7 (NV ctrl), *n* = 3–8 (LTV ctrl), *n* = 3–7 (HTV ctrl), *n* = 2–7 (LTV ATB), or *n* = 5–6 (HTV ATB). Analysis was by two-way analysis of variance/Tukey’s multiple comparison test; ^#^difference in HTV ATB vs. HTV Ctrl; *as indicated, *^/#^*P* < 0.05, **^/##^*P* < 0.01, ***P < 0.001, ****P < 0.0001

**Fig. 5:**
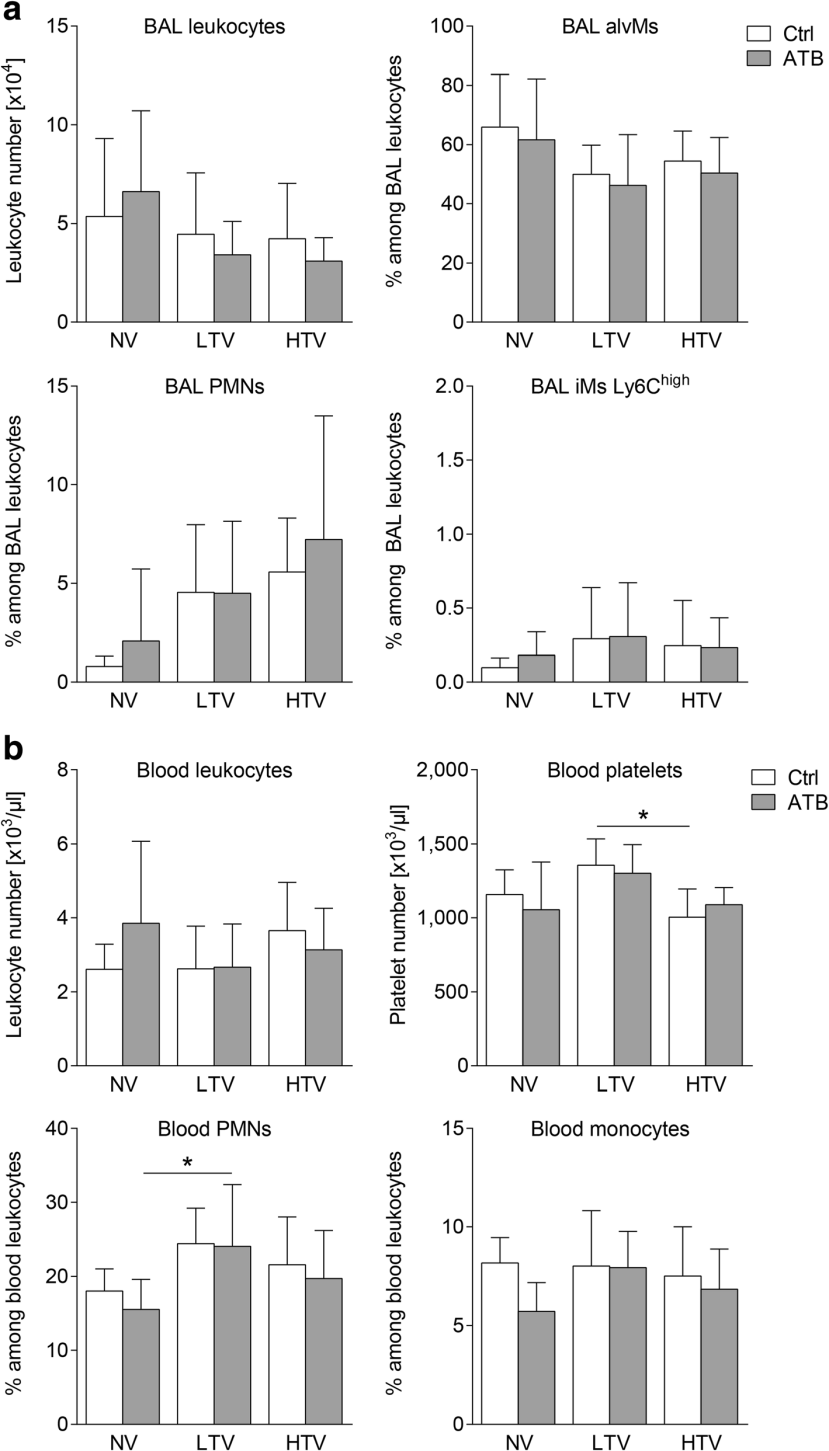
Microbiota depletion prior to mechanical ventilation had no impact on composition and recruitment of bronchoalveolar lavage (BAL) innate alveolar cells and blood leukocyte populations upon ventilation. Mice received antibiotic broad-spectrum therapy (ATB). Control (Ctrl) mice did not receive antibiotic treatment. Ventilator-induced lung injury was induced 3 days after completing the antibiotic treatment protocol by high tidal volume (HTV) ventilation (34 ml/kg; positive end-expiratory pressure = 2 cmH_2_O, 4 h). Additional groups of mice were ventilated with low tidal volume (LTV) or did not receive ventilation (NV). **a** Numbers of BAL leukocytes and frequencies of alveolar macrophages (alvMs), polymorphonuclear cells (PMNs) and inflammatory macrophages (iMs) Ly6C^high^ in BAL as quantified by flow cytometry. **b** Numbers of blood leukocytes and platelets and frequencies of PMNs (granulocytes minus eosinophils) and monocytes measured in EDTA-blood by Scil Vet abc hematology analyzer. Values are given as mean and SD. In **a** leukocyte numbers, *n* = 7 (NV ctrl, NV ATB, LTV ctrl, HTV ATB), *n* = 6 (HTV ctrl) or *n* = 3 (LTV ATB); leukocyte frequencies, *n* = 9 (NV ATB), *n* = 8–9 (NV ctrl), *n* = 8 (LTV ctrl, HTV ctrl, HTV ATB), or *n* = 6–7 (LTV ATB); in **b** *n* = 9 (NV ctrl, NV ATB), *n* = 8 (HTV ATB), or *n* = 7 (LTV ctrl, LTV ATB, HTV ctrl). Analysis was by two-way analysis of variance/Tukey multiple comparison test, *P < 0.05

To evaluate the effect of microbiota depletion on the inflammatory response to MV, we measured protein and mRNA levels of key inflammatory mediators and assessed various innate and adaptive immune cell populations in the blood, lungs, and alveolar spaces. Mice receiving antibiotic therapy prior to HTV-ventilation had higher CXCL1/keratinocyte-derived chemokine (KC), and IL-6 protein levels in lung tissue, whereas protein levels in plasma from HTV-ventilated mice were not increased after microbiota depletion (Fig. 4a, b). Examination of gene regulation after antibacterial therapy revealed enhanced *Il6* mRNA expression in the lungs of HTV-ventilated mice that received antibiotic therapy prior to ventilation (Fig. 4c). Notably, despite increasing levels of inflammatory molecules, microbiota depletion prior to MV had no significant impact on recruitment of inflammatory cells (polymorphonuclear leukocytes (PMN) and inflammatory macrophages (iMs)) into the alveolar spaces and the lungs in HTV-ventilated mice. Likewise, further cellular populations (including CD4 T cells, CD8 T cells, regulatory T cell (Treg), γδ T cells, alveolar macrophages (alvMs), blood monocytes, blood granulocytes, platelets) remained unaffected by antibacterial treatment prior to ventilation (Fig. 5 and Additional file 1: Figure S5).

## Discussion

In the current study we found that disruption of the microbiota by antibiotic therapy prior to mechanical ventilation (MV) increased the susceptibility to ventilator-induced lung injury (VILI). Mechanical ventilation is a life-saving intervention without an alternative for patients with respiratory failure, but it may aggravate or even induce VILI [29]. Further, mechanically ventilated critically ill patients are frequently exposed to antibiotic treatment (reviewed in [30]) or receive selective digestive decontamination (SDD) [31]. Antibiotics change the microbiota composition, which impacts the local and systemic inflammatory response towards various stimuli [32–36]. Taking this into account it is of great interest to determine the effect of antibiotic therapy and associated microbiota alteration on the development of VILI.

To investigate this interaction, we treated mice for 6–8 weeks with a broad-spectrum antibiotic regimen to generate mice with a virtually depleted gut microbiota. Using the described cocktail, we showed a significant decline in relevant bacterial species in the gut, but as part of the antibiotic applied is absorbed in the gut, the general microbiome of the animals is likely to be changed, which has to be considered in the interpretation of our results. It has been reported that treatment with antibiotics renders patients susceptible to pulmonary infections including multidrug-resistant pathogens [37, 38]. Recently, we have shown that antibiotic treatment lowers pulmonary IgA production and enhances susceptibility of mice towards *P. aeruginosa* [12]. However, the impact of the microbiota on the development of a hyperinflammatory sterile lung injury like VILI remains unclear. Using the described antibiotic cocktail including systemically effective antibiotics, we efficiently depleted the microbiota of the gut, but probably also of other organs including the lungs.

Prolonged antibiotic treatment prior to ventilation significantly aggravated VILI, as indicated by increased pulmonary permeability, decreased pulmonary compliance reflecting lung edema, and macroscopic signs of aggravated lung injury. An extensive proinflammatory response towards MV is a major driver of VILI, and alteration of the microbiota was reported to have a significant impact on the regulation of pulmonary inflammation [39, 40]. We found elevated protein levels of KC and IL-6 in antibiotic-treated mice subjected to VILI, but did not detect any effect of microbiome depletion on the inflammatory response in non-ventilated mice. It remains unclear if the aggravated inflammatory response to VILI in antibiotic-treated mice may be the consequence of aggravated lung injury due to MV and associated inflammation or if the absence of an intact microbiome prior to VILI may have rendered the lung more reactive leading to an increased inflammatory response.

In this study, we implemented severely injurious ventilation in mice applying a tidal volume of 34 ml/kg, which was much higher than the standard of lung-protective ventilation tidal volume of 6 ml/kg in humans with acute respiratory distress syndrome (ARDS). At first view, this might outrange the stress and strain applied during MV in patients with ARDS. However, functional residual lung capacity in ARDS is severely reduced, which is referred to as the “baby lung” in patients with ARDS [41]. Even with a tidal volume of 6 ml/kg it is unclear how much lung strain is produced during MV but available data show that lung stress and strain are significantly increased [42]. Further, the high grade of tissue inhomogeneity in lungs in ARDS, in which open, atelectatic, and collapsed but recruitable lung areas coexist, locally results in lung stress exceeding the measured airway pressure by far [43]. In the applied model, we used previously healthy lungs, sufficiently recruited at the beginning of MV. Therefore, the lungs are lacking consolidations and relevant atelectatic lung regions that account for the reduced functional residual capacity resulting in elevated lung strain in patients with ARDS even under lung-protective ventilation. Further, in the recruited healthy lungs homogenously ventilated local lung stress is minimized. Therefore simulating “clinically relevant” lung stress and strain accruing in patients with ARDS requires an experimental approach that provokes significant lung stress/strain in healthy lungs with previously normal residual capacity and in the context of homogenously open and ventilated lung tissue. Moreover, the lung injury to be investigated has to be prevalent in a feasible timeframe. Taking all this into account, ventilation with 34 ml/kg in healthy mice is a reasonable experimental approach for our study meeting the American Thoracic Society (ATS) criteria for lung injury research in animal models [21].

The complex interaction between gut and lung is still not fully understood. One possible pathway encompasses altered communication between these two compartments over the “gut-lung axis” (reviewed in [44, 45]). For instance, the risk of developing airway diseases is increased after antibiotic-induced alterations in the gut microbiota in early childhood [46–48]. The intestinal microbiota might influence pulmonary immune responses by distributing bacterial metabolites via the bloodstream, which may have direct immunomodulatory effects [14, 49]. One of these immunomodulating mediators might be short-chain fatty acids (SCFA) which result from digestion and converting of indigestible nutrients by the commensal bacteria and have been shown to have anti-inflammatory action (as reviewed by Marsland [14]). Zhang et al. showed that prophylactic administration of the SCFA sodium butyrate reduced lung injury after sepsis and attenuated lung lesions [50]. Reduced anti-inflammatory properties could be a possible explanation for the aggravated inflammatory response to VILI that we observed. However, further studies are warranted to dissect in the current context whether elevated cytokines mediate the aggravation of lung injury or result from ventilation-induced tissue injury leading e.g. to the release of proinflammatory DAMPs [51, 52].

The approach of using long-term broad-spectrum antibiotics is a well-established method to deplete the microbiome in mice with no reports of any remarkable toxic effect to the animals [20, 26–28]. Accordingly, histopathological analysis of naive lungs in microbiota-depleted mice in our study did not reveal any signs of pulmonary toxicity of the applied antibiotic regimen. To minimize a possible influence of circulating bacterial components arising after antibiotic lysis of bacteria, we stopped the antibiotic therapy 3 days prior to ventilation to “wash out” all possible remaining antibiotic substances or circulating bacterial metabolites. Taking this into account, an underlying toxic effect of the antibiotics on the lung tissue is very unlikely.

Nevertheless, as yet unknown effects of such antibiotic intervention might have influenced the results of this study. Studies using germ-free mice and mice recolonized after antibiotic treatment may offer further insights and allow to dissect if the microbiome itself or determine whether other potential antibiotic-related effects have affected the susceptibility to VILI. Thus, despite all limitations of an experimental animal model, we judge the complex model used in the present study to be suitable to examine the influence of the microbiota on the development of lung injury, best modeling the situation of ICU patients.

## Conclusions

In summary, microbiota depletion by antibacterial therapy prior to MV enhanced inflammatory responses towards MV and rendered mice susceptible to the development of VILI. The findings highlight a potentially relevant interaction of the microbiome with the inflammatory response in lung injury.

## Additional file

Additional file 1: **Figure S1.** Intestinal microbiota density declines after oral antibiotic treatment. **Figure S2.** Mean arterial pressure during mechanical ventilation. **Figure S3.** Exemplary flow cytometric gating strategy of innate immune cell populations in the alveolar spaces. **Figure S4.** Antibiotic therapy did not per se lead to lung injury as assessed histologically. **Figure S5.** Microbiota depletion prior to mechanical ventilation had no impact on composition and recruitment of innate and adaptive alveolar cells in the lungs. **Table S1.** Primers used for qPCR. (PDF 1720 kb)

## Abbreviations

ATB: Antibiotic-treated
alvMs: Alveolar macrophages
ANOVA: Analysis of variance
ARDS: Acute respiratory distress syndrome
BAL: Bronchoalveolar lavage
BALF: Bronchoalveolar lavage fluid
C_stat_: Static compliance
Ctrl: Control
E: Dynamic elastance
ELISA: Enzyme-linked immunosorbent assay
FiO_2_: Inspiratory fraction of oxygen
HSA: Human serum albumin
HTV: High tidal volume
IL-1β: Interleukin-1 beta
IL-6: Interleukin-6
iMs: Inflammatory macrophages
KC: Keratinocyte-derived chemokine
LTV: Low tidal volume
mAbs: Monoclonal antibodies
MAP: Mean arterial pressure
MIP-1α: Macrophage inflammatory protein-1α
MV: Mechanical ventilation
NV: Non-ventilated
OI: Oxygenation index
paCO_2_: Partial arterial pressure of carbon dioxide
paO_2_: Partial arterial pressure of oxygen
PBS: Phosphate-buffered saline
PEEP: Positive end-expiratory pressure
PMNs: Polymorphonuclear leukocytes
qPCR: Real-time polymerase chain reaction
SCFA: Short-chain fatty acids
Treg: Regulatory T cells
VILI: Ventilator-induced lung injury
