# Supplementary material for: Ventilator-induced lung injury is aggravated by antibiotic mediated microbiota depletion in mice
Source: Crit Care. 2018 Oct 29;22:282. doi: 10.1186/s13054-018-2213-8 (PMC6206919; doi:10.1186/s13054-018-2213-8)
Supplement: Supplementary file 1 — Figure S1. Intestinal microbiota density declines after oral antibiotic treatment. Figure S2. Mean arterial pressure during mechanical ventilation. Figure S3. Exemplary flow cytometric gating strategy of innate immune cell populations in the alveolar spaces. Figure S4. Antibiotic therapy did not per se lead to lung injury as assessed histologically. Figure S5. Microbiota depletion prior to mechanical ventilation had no impact on composition and recruitment of innate and adaptive alveolar cells in the lungs. Table S1. Primers used for qPCR. (PDF 1720 kb) [file 13054_2018_2213_MOESM1_ESM.pdf]

Supplementary Figures

Figure S1. Intestinal microbiota density declines after oral antibiotic treatment. Mice received antibiotic broad-spectrum therapy (ATB). Control (Ctrl) mice did not receive antibiotic treatment. Feces samples were collected and stored at -80°C until analysis. 16s rRNA of intestinal bacterial groups including total eubacterial load (TL), *Enterobacteriaceae* (EB)*, Enterococcus* spp. (EC)*,* Lactic acid bacteria (LB), *Bifidobacterium* spp. (BB), *Bacteroides/Prevotella spp.* (BP), *Clostridium coccoides* group (CC), *Clostridium leptum* group (CL), *Mouse Intestinal Bacteroides (MIB*) were separately detected, quantified and are expressed as gene copies per ng DNA*.* Samples were pooled per group (antibiotic-treated vs. control) for analysis. Scatter dot plot with single values (n = 15 each group) and median, Mann Whitney *U*-test, **P* <0.05, *****P* <0.0001.

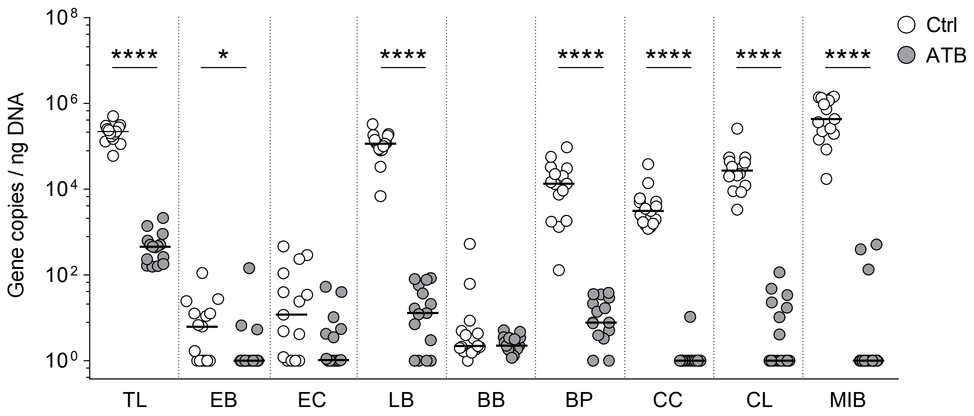

Figure S2. Mean arterial pressure during mechanical ventilation. Mice received antibiotic broad-spectrum therapy (ATB). Control (Ctrl) mice did not receive antibiotic treatment. VILI was induced three days after completing antibiotic treatment protocol by high tidal volume (HTV) ventilation (34 ml/kg; PEEP = 2 cmH_2O, 4 h). Additional groups of mice were ventilated with low tidal volume (LTV) or did not receive ventilation (NV). Mean arterial pressure (MAP) was measured every 10 minutes. Values are given as mean ± SD, n = 11 (LTV ctrl), 10 (HTV ctrl) or 9 (HTV ATB) or 6 (LTV ATB), 2-way repeated measures ANOVA/Tukey's multiple comparison test.

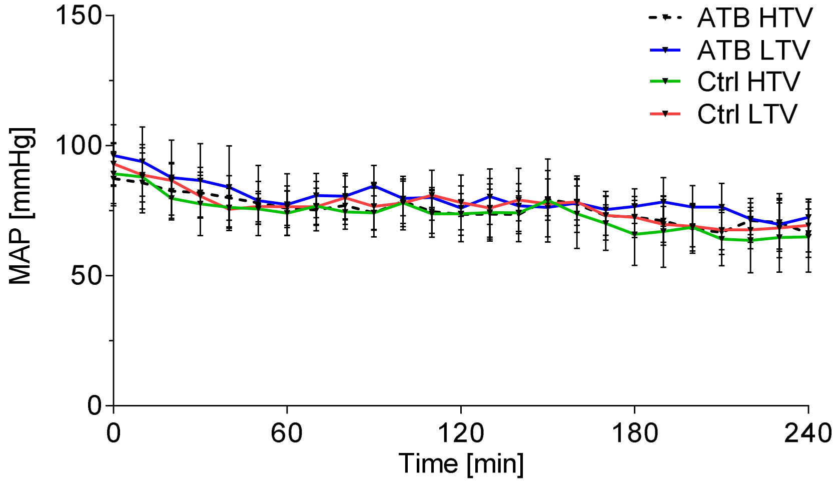

Figure S3. Exemplary flow cytometric gating strategy of innate immune cell populations in alveolar spaces. Representative dot blots illustrating cytometric gating strategy of innate immune cell populations in BAL and lungs. AlvMs, alveolar macrophages; PMN, polymorphonuclear leukocytes; iMs Ly6C^{high}, inflammatory macrophages.

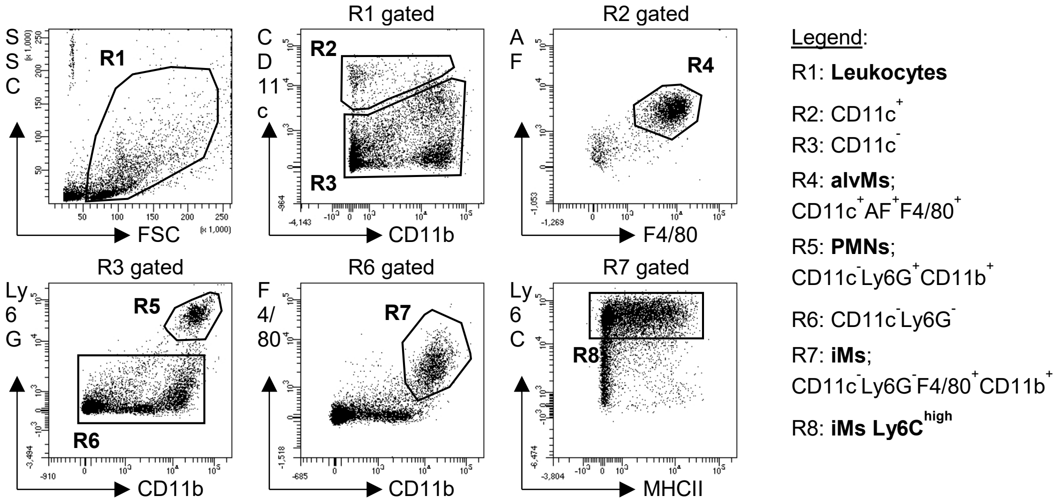

Figure S4. Antibiotic therapy did not per se lead to lung injury as assessed histologically. Mice received antibiotic broad-spectrum therapy (ATB). Control (Ctrl) mice did not receive antibiotic treatment. In Hematoxylin-eosin (**a**) and PAS (**b**) stained lung sections of non-ventilated mice (NV), no signs of toxic lung injury were observed. Representative images are shown (n = 3 each group), scale bar 50 µm (valid for all photomicrographs).

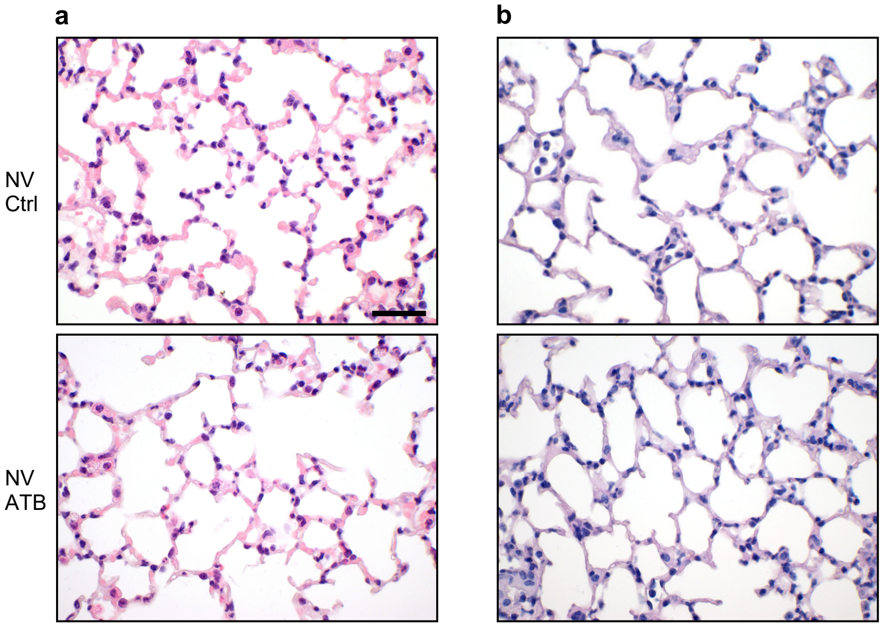

Figure S5. Microbiota depletion prior to mechanical ventilation had no impact on composition and recruitment of innate and adaptive alveolar cells in lungs. Mice received antibiotic broad-spectrum therapy (ATB). Control (Ctrl) mice did not receive antibiotic treatment. VILI was induced three days after completing antibiotic treatment protocol by high tidal volume (HTV) ventilation (34 ml/kg; PEEP = 2 cmH_2O, 4 h). Additional groups of mice were ventilated with low tidal volume (LTV) or did not receive ventilation (NV). **a**, Numbers of lung leukocytes and frequencies of alveolar macrophages (alvMs), polymorphonuclear leukocytes (PMN) and inflammatory macrophages (iMs Ly6C^{high}) in lung as quantified by flow cytometry. **b**, Frequencies of lymphocytes, CD3$^+$ T cells and $\gamma\delta$ T cells in lung as quantified by flow cytometry. Values are given as mean and SD, (a, leukocyte number), n = 8 (LTV ctrl), 7 (NV ctrl, NV ATB), 6 (HTV ATB), 5 (LTV ATB) and 4 (HTV ctrl) or (a, leukocyte frequencies; and b), n = 8-9 (NV ctrl, NV ATB), 8 (LTV ctrl), 7-8 (HTV ctrl, HTV ATB) or 6-7 (LTV ATB), 2-way ANOVA/Tukey´s multiple comparison test, ****P <0.0001 vs. NV Ctrl, $^{\#}P$ <0.05, $^{\#\#\#\#}P$ <0.0001 vs. NV ATB.

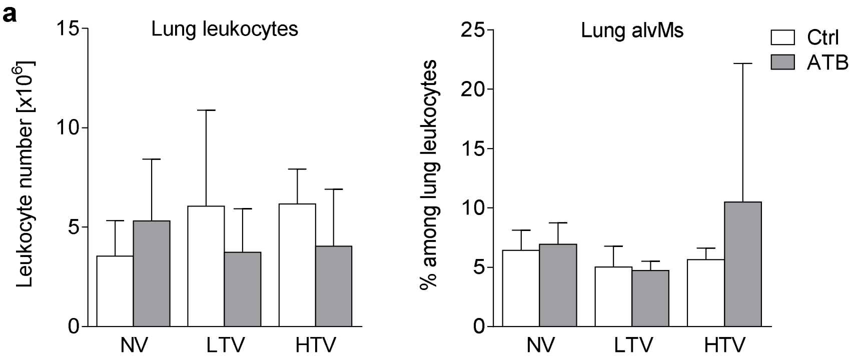

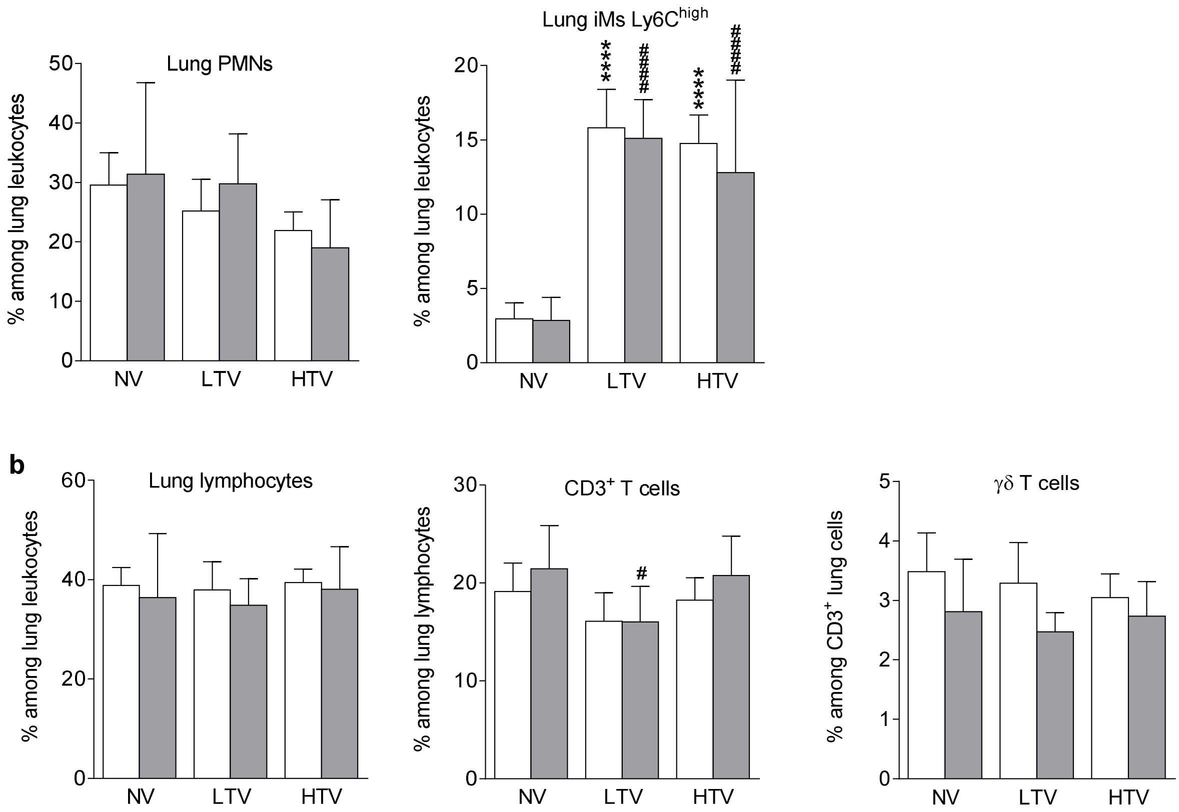
Lung PMNs
% among lung leukocytes
0
10
20
30
40
50
NV
LTV
HTV
Lung iMs Ly6C^high
% among lung leukocytes
0
5
10
15
20
****
####
****
####
b
Lung lymphocytes
% among lung leukocytes
0
20
40
60
CD3+ T cells
% among lung lymphocytes
0
10
20
30
#
γδ T cells
% among CD3+ lung cells
0
1
2
3
4
5

Supplementary Table

Table S1. Primers used for qPCR

gene	sequence		accession number
Ccl-2	forward	GCATCTGCCCTAAGGTCTTCA	NM_011333
	reverse	GTGGAAAAGGTAGTGGATGCATT	
	probe	FAM-ACCTTTGAATGTGAAGTTGACCCGTAAATCTGAAG-TAMRA	
Il-6	forward	CCACGGCCTTCCCTACTTC	NM_031168
	Reverse	TGCACAACTCTTTTCTCATTTCCA	
	Probe	FAM-TCACAGAGGATACCACTCCCAACAGACCTG-TAMRA	
Gapdh	forward	TGTGTCCGTCGTGGATCTGA	NM_008084
	reverse	CCTGCTTCACCACCTTCTTGA	
	probe	FAM-CCGCCTGGAGAAACCTGCCAAGTATG-TAMRA	
Il-1β	purchased from Applied Biosystems<br>Assay no.: Mm00434228		NM_008361
Il-10	purchased from Applied Biosystems<br>Assay no.: Mm00439614		NM_010548
